# The complete plastome sequence of *Illigera grandiflora*

**DOI:** 10.1080/23802359.2021.1951625

**Published:** 2021-07-15

**Authors:** Yaya Qu, Linyi Yang, Zhenghai Sun, Luyao Ma, Jing Xin, Yu Song, Peiyao Xin

**Affiliations:** a Southwest Research Center for Landscape Architecture Engineering, State Forestry and Grassland Administration, Southwest Forestry University, Kunming, PR China; b Yunnan Province South and Southeast Asia Joint R&D Center of Economic Forest Full Industry Chain, Southwest Forestry University, Kunming, PR China; c Key Laboratory of Forest Resources Conservation and Utilization in the Southwest Mountains of Ministry of Education, Southwest Forestry University, Kunming, PR China; d Center for Integrative Conservation, Xishuangbanna Tropical Botanical Garden, Chinese Academy of Sciences, Jinghong, PR China

**Keywords:** *Illigera*, plastome, phylogenetic relationship

## Abstract

*Illigera grandiflora*, a kind of traditional medicinal liana, belongs to the *Illigera* Blume of the Hernandiaceae. In this study, we reported the characteristics of complete plastome for *I. grandiflora*. Its total plastome was 156,138 bp in length, comprising a large single-copy region(LSC) of 84,931 bp, a small single-copy region (SSC) of 18,544 bp, and a pair of inverted repeat (IR) regions of 26,549 bp. The overall GC content was 39.16% (LSC, 37.77%; SSC, 33.89%; IR, 43.21%). The plastome encoded 134 genes, including 83 protein-coding genes, 42 transfer RNA genes, and 10 ribosomal RNA genes. The relationships in our phylogeny showed that the two *Illigera* species are located in the same clade, with *Hernandia nymphaeifolia* being the next sister group, followed by *Wilkiea huegeliana*.

*Illigera grandiflora* W.W.Sm. & Jeffrey, an evergreen liana with 2–6 m tall, inhabits forests at an altitude of 800–2100 m and is widely distributed in India, north Myanmar, and southwestern China (Chinese Flora Editorial Board, Chinese Academy of Sciences 2008). The root and stem of *I. grandiflora* was used to treat dropsy and traumatic injury (Gao 2007; Huang 1985), and previous studies have revealed the major chemical components of the plants of the genus *Illigera* Blume are alkaloids and terpenoids. Li et al. (2019) have isolated a new dibenzopyrrocoline alkaloid, together with five known ones from *I. grandiflora*, three of them exhibited the moderate inhibitory activity against acetylcholinesterase (AChE) or butyrylcholinesterase (BChe), it has showed great medical potential. But there are few studies of *I. grandiflora* on genomic at present. Recent studies (Xin et al. 2020) have finished the complete chloroplast genomes sequencing of *Illigera celebica* (LAU199). In order to compare the chloroplast differences among different species and to better understand its phylogenetic relationships between them and other Laurales species, we reported the characteristics of complete plastome for *I. grandiflora*, and then reconstructed a phylogenetic tree.

Fresh leaves of *I. grandiflora* were collected from Cangyuan Wa Autonomous County, Yunnan, China: (23.2°N, 99.4°E) for genomic DNA extraction using modified CTBA method (Cai et al. 2014), and was then sequenced by Illumina Hiseq 2000 platform at BGI-Shenzhen. A specimen was deposited at XTBG’s Biodiversity Research Group (contact Song-Yu, songyu@xtbg.ac.cn) under the voucher number SY5852. Aligning, assembly (reference sequences are LAU00199 and MN990581), and annotation were conducted by MAFFT v.7 online program (https://mafft.cbrc.jp/alignment/server/) (Katoh and Standley 2013), GetOrganelle software (Jin et al. 2018), and Geneious R8.1.3 (Kearse et al. 2012) respectively. The plastid genome phylogenetic relationships were reconstructed based on a maximum-likelihood (ML) analysis with the GTR + F + R2 model by iqtree version 1.6.7.1 program using 1000 bootstrap replicates (Nguyen et al. 2015).

The plastome of *I. grandiflora* (MW755975) is a circular DNA molecule with a length of 156,138 bp, which is 15 bp larger than *Illigera celebiea* (LAU00199). The complete plastome contains a large singlecopy region (LSC, 84,931 bp), a small single-copy region (SSC, 18,544 bp), and a pair of inverted repeats (IRs, 26,549 bp). The overall GC content is 39.16%, the corresponding values of the LSC, SSC,and IR regions are 37.77%, 33.89%, and 43.21% respectively. The plastome encoded a set of 120 genes, of which 76 are protein‐coding genes, 36 are transfer RNA genes, and 8 are rRNA genes.

The ML tree was built on complete plasomes of 22 related species, *Liriodendron chinense* (KU170538) was treated as the out-group (Figure 1). The phylogenetic tree was divided into three mian clades corresponding to four families: Hernandiaceae, Monimiaceae, Lauraceae, and Calycanthaceae. Most relationships had high internal support. Phylogenetic analysis based on all plastomes supported that sisterhood of *I. grandiflora* and *I. celebiea*, with *H. nymphaeifolia* (MG838431) being the next sister group, followed by *W. huegeliana* (KT716505). In addition, we reconfirmed the sisterhood of Lauraceae and a clade containing Hernandiaceae and Monimiaceae (Song et al. 2020).

**Figure 1.**
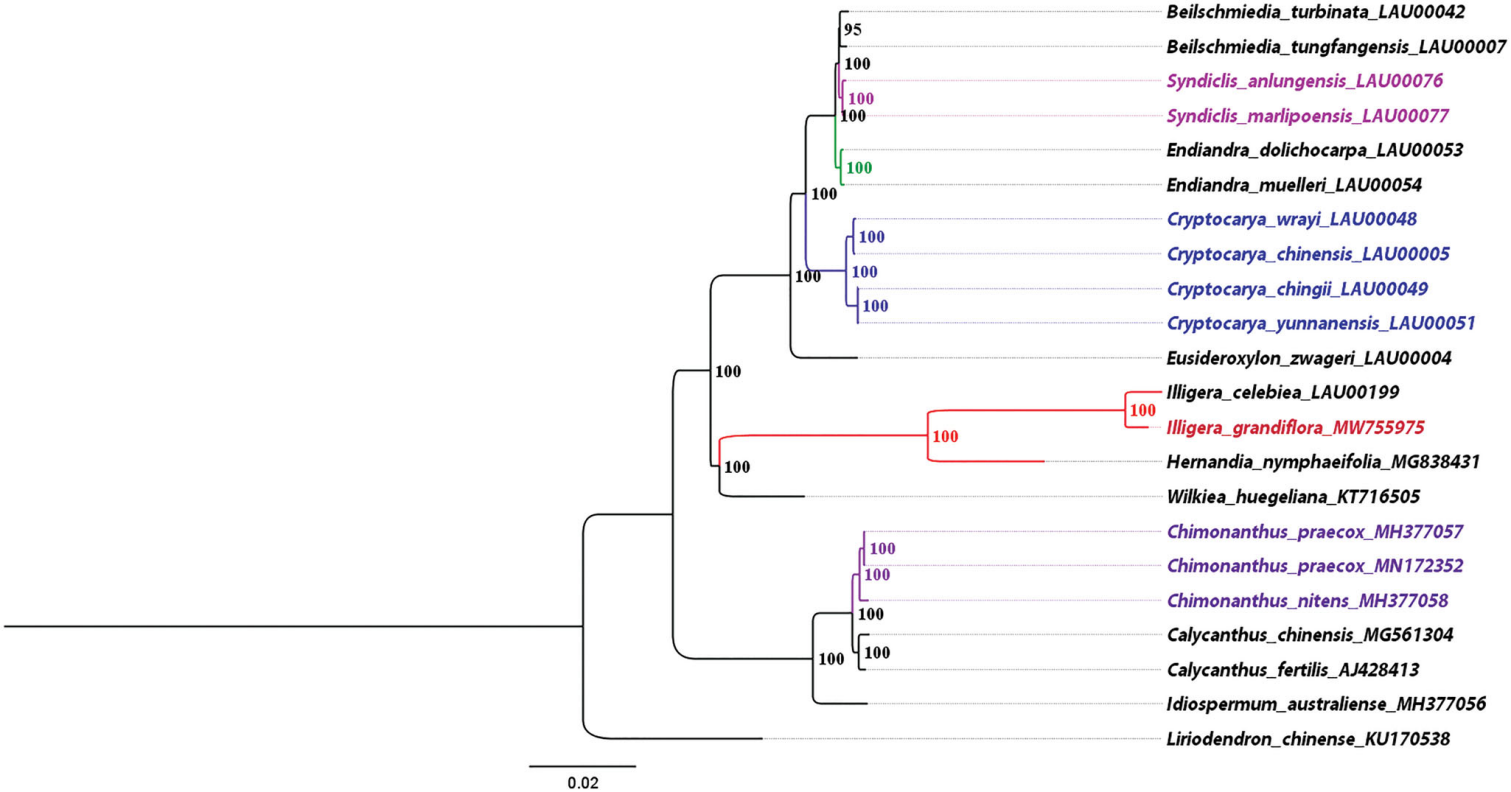
The ML phylogenetic tree for *I. grandiflora* based on other 21 species (11 in Lauraceae, 2 in Hernandiaceae, 1 in Monimiaceae, 6 in Calycanthaceae, and 1 in Magnoliaceae) plastid genomes; the complete plastome sequences were from Lauraceae Chloroplast Genome Database (https://lcgdb.wordpress.com/) (13 species those numbers ending with LAU) and NCBI (other 9 species).

## Data Availability

The genome sequence data that support the findings of this study are openly available in GenBank of NCBI at (https://www.ncbi.nlm.nih.gov/) under the accession no. MW755975. The associated BioProject, SRA, and Bio-Sample numbers are PRJNA715651, SAMN18388833, and SRR14018806, respectively.
